# Correction: Alternative method to visualize receptor dynamics in cell membranes

**DOI:** 10.1371/journal.pone.0308231

**Published:** 2024-07-29

**Authors:** Cosetta Ravelli, Michela Corsini, Anna Ventura, Mattia Domenichini, Elisabetta Grillo, Stefania Mitola

The first and last names of the authors are incorrectly switched. The correct author names are:

Cosetta Ravelli, Michela Corsini, Anna Ventura, Mattia Domenichini, Elisabetta Grillo, Stefania Mitola. The correct citation is: Ravelli C, Corsini M, Ventura A, Domenichini M, Grillo E, Mitola S (2024) Alternative method to visualize receptor dynamics in cell membranes. PLoS ONE 19(6): e0304172. https://doi.org/10.1371/journal.pone.0304172
