# Supplementary material for: Alternative method to visualize receptor dynamics in cell membranes
Source: PLoS One. 2024 Jun 11;19(6):e0304172. doi: 10.1371/journal.pone.0304172 (PMC11166301; doi:10.1371/journal.pone.0304172)
Supplement: S1 File — (PDF) [file pone.0304172.s001.pdf]

Ravelli C et al., **Supporting information (S1)**

Step-by-step protocol

1 Cell transfection (day 1 and 2)

- The day before transfection, seed 2x104/cm2 of CHO cells in 6 well plate in F12 medium supplemented with 100 IU/mL penicillin and 100 µg/mL streptomycin and 10% FCS (complete medium) and culture under normal conditions at 37 °C in 5% CO_2. When using different cell lines, ensure a cell density to allow 60-70% of confluence the day of the transfection.
- After 24 hours, transfect each well of CHO cells with 4 µg of pBE-hVEGFR2-eYFP and 8 ng of PEI (1µg/µL) in serum and antibiotics free F12 medium. After 4 hours, replace medium with complete medium.

2 Protein immobilization to glass coverslip (day 3)

- Incubate the 2 well chambered glass coverslips with 100 µL of sterile PBS containing 2 µg/mL of human VEGF-A for 16 hours at 4°C. The coating is carried out by placing a drop containing the recombinant protein in the center of the well in order to obtain a 10 mm diameter coating spot. VEGF-A can be replaced by other ligands able to recruit other specific receptors.
- After 16 hours, remove unbound ligand and wash the coverslips 3 times with cold and sterile PBS.
- Block nonspecific binding sites with 1 mg/mL of BSA for 1 hour at room temperature.

Under these conditions, ligand binds to the coverslip in a dose-dependent manner, with maximal binding at coating concentrations ≥ 2 µg/mL. Using this concentration, it is possible to have a spot with a high concentration of ligands.
Substratum-immobilized ligand is resistant to high molar salt (2 mol/L NaCl) and detergent (0.2% Triton X-100) washes [1,2.

3 Cell preparation (day 3)

- Put glass coverslips on the bottom of a 24 well plate and ensure it remains to the bottom of the well while seeding the cells.
- 24 hours after cell transfection, plate CHO at the density of 75.000/cm2 in complete medium on the coverslips and culture under normal conditions for 16 hours. When using different cell lines, ensure a cell density to allow 50-80% of confluence the day of image acquisition.

4 Image acquisition (day 4)

- Replace the complete medium of transfected cells with F12 (without phenol red) 1% FCS and culture under normal conditions for 2 hours.
- After 2 hours of starvation, flip upside-down the cell-plated coverslips on immobilized-VEGF chambered in F12 1% FCS.
- Put the sample in the microscope incubator at 37 °C and 5% CO_2.

To analyze VEGFR2 recruitment we acquired Z-stack images for 120 minutes.

- Acquire images using YFP fluorescence filter set (excitation: 500/20; dichroic: long pass 512; emission: 535/30).

CRITICAL STEP: It is very important to acquire all the images with the same camera exposure. This allows you to compare different images or different experimental conditions, when necessary.

- Acquire imaging with a PlanApochromat 63X/1.4NA Oil objective and Apotome structured illumination that allow a sectioning of 1.3 µm. Set an overlap of 50% between two consecutive stacks. On average, a whole CHO cell is acquired in 10-12 slices, with a total thickness of acquisition of 13-15.6 µm.
- Process images without deconvolution.

5 Image quantification and data analysis (day 5)

- Open image series in Fiji as hyperstacks. A sequence of images open, each representing a stack.
- Convert image stack in 8 bits.
- Adjust brightness and contrast in order to clearly see cells in each stack.

Note that any adjustment in brightness and contrast for the visualization won't modify fluorescence quantification.

- In Analyze > Set Measurements select Area and Area percentage options.
- Open the Threshold menu and set threshold manually in order to clearly see the specific fluorescence standing out from the background.

CRITICAL STEP: It is very important to set the same threshold for all the images. This allows you to compare different images or different experimental conditions, when necessary.

- Draw, using freehand selection, the projection of the cell. Analyze one cell at a time. Scroll through the image sequence measuring Area and Area percentage in every stack.
- Save data for the analysis.
- Calculate the number of pixels positive for VEGFR2 associated fluorescence using the formula:

N° of VEGFR2-positive pixels = Area percentage * (Area/100)

- Sum all the pixel from each Z-stack to obtain the total amount of VEGFR2-positive pixels for cell
- Calculate the distribution of VEGFR2 in each stack using the formula:

% of VEGFR2 area = (N° of VEGFR2-positive pixels / total amount of VEGFR2-positive pixels) * 100

Note that the sum of % of VEGFR2 area from all the stacks should be 100.

6 3D reconstruction

- Select a region of interest (ROI) that includes one cell or more. Save the image.
- Create orthogonal projection by choosing, from Image > Stacks the Orthogonal Views command.
- Create a 3D image using "3D viewer " plugin.

References

1. Ravelli C, Grillo E, Corsini M, Coltrini D, Presta M, Mitola S (2015) beta3 Integrin Promotes Long-Lasting Activation and Polarization of Vascular Endothelial Growth Factor Receptor 2 by Immobilized Ligand. Arteriosclerosis, thrombosis, and vascular biology 35 (10):2161-2171. doi:10.1161/ATVBAHA.115.306230
2. Andres G, Leali D, Mitola S, Coltrini D, Camozzi M, Corsini M, Belleri M, Hirsch E, Schwendener RA, Christofori G, Alcami A, Presta M (2009) A pro-inflammatory signature mediates FGF2-induced angiogenesis. Journal of cellular and molecular medicine 13 (8B):2083-2108. doi:10.1111/j.1582-4934.2008.00415.x
