# Supplementary material for: Alternative method to visualize receptor dynamics in cell membranes
Source: PLoS One. 2024 Jun 11;19(6):e0304172. doi: 10.1371/journal.pone.0304172 (PMC11166301; doi:10.1371/journal.pone.0304172)
Supplement: Attachment — Submitted filename: rebuttal.pdf [file pone.0304172.s002.pdf]

UNIVERSITÀ DEGLI STUDI DI BRESCIA
DIPARTIMENTO DI MEDICINA MOLECOLARE E TRASLAZIONALE
SEZIONE DI BIOTECNOLOGIE
Prof.ssa Stefania Mitola
Tel. 030 3717312 - E-Mail: stefania.mitola@unibs.it

Brescia, 18/4/2024

Dear Prof. Eleftherios Paschalis Ilios,

Many thanks for your correspondence on our manuscript entitled "Alternative method to visualize receptor dynamics in cell membranes" by C. Ravelli et al. (PONE-D-24-04439). Taking advantage of comments and suggestions of the Reviewers, we modified our previous version that we would like to submit to your attention.

We have addressed reviewers' criticisms (*in italics*) as it follows:

1. Does the manuscript report a protocol which is of utility to the research community and adds value to the published literature?
Reviewer #1: Yes
Reviewer #2: Yes

We thank reviewers for appreciating the utility of our protocol and for recognizing its novelty.

2. Has the protocol been described in sufficient detail?
To answer this question, please click the link to protocols.io in the Materials and Methods section of the manuscript (*DOI: dx.doi.org/10.17504/protocols.io.rm7vzj8z8lx1/v1 (Private link for reviewers: https://www.protocols.io/private/46322724FDB111EEB7ED0A58A9FEAC02 to be removed before publication.)*

) or consult the step-by-step protocol in the Supporting Information files. The step-by-step protocol should contain sufficient detail for another researcher to be able to reproduce all experiments and analyses.
Reviewer #1: Partly
Reviewer #2: Yes

Following the Editor suggestion the step-by-step protocol, included in Supporting information in the previous version, was included and published into protocols.io . Please see:
Please see DOI: dx.doi.org/10.17504/protocols.io.rm7vzj8z8lx1/v1 (Private link for reviewers: https://www.protocols.io/private/46322724FDB111EEB7ED0A58A9FEAC02 to be removed before publication.)

3. Does the protocol describe a validated method?
The manuscript must demonstrate that the protocol achieves its intended purpose: either by containing appropriate validation data, or referencing at least one original research article in which the protocol was used to generate data.
Reviewer #1: Yes
Reviewer #2: Yes

Here we described immobilized VEGF-A-recruited receptor to demonstrate the cogency of our method. The method allowed us to compare different experimental conditions such as different stimuli, inhibitors, receptors mutants and multiple receptors and co-receptors at the same time (see Damioli et al.).

4. If the manuscript contains new data, have the authors made this data fully available?

UNIVERSITÀ DEGLI STUDI DI BRESCIA
DIPARTIMENTO DI MEDICINA MOLECOLARE E TRASLAZIONALE
SEZIONE DI BIOTECNOLOGIE
Prof.ssa Stefania Mitola
Tel. 030 3717312 - E-Mail: stefania.mitola@unibs.it

The PLOS Data policy requires authors to make all data underlying the findings described in their manuscript fully available without restriction, with rare exception (please refer to the Data Availability Statement in the manuscript PDF file). The data should be provided as part of the manuscript or its supporting information, or deposited to a public repository. For example, in addition to summary statistics, the data points behind means, medians and variance measures should be available. If there are restrictions on publicly sharing data—e.g. participant privacy or use of data from a third party—those must be specified.
Reviewer #1: N/A
Reviewer #2: Yes
We added statistic detail s in figure captions.

5. Is the article presented in an intelligible fashion and written in standard English?
PLOS ONE does not copyedit accepted manuscripts, so the language in submitted articles must be clear, correct, and unambiguous. Any typographical or grammatical errors should be corrected at revision, so please highlight any specific errors that need correcting in the box below.
Reviewer #1: Yes
Reviewer #2: Yes

The typos and grammar errors were corrected.

Reviewer Comments to the Author:

Reviewer #1

The proposed manuscript by R. Cosetta and al. is dedicated to the development of a new approach to determine the receptor dynamics in the cell membrane. This approach was developed to use technology easily available in most research centers. This technological manuscript well describes the interest of such approach.
However, some important points should be addressed to improve this manuscript:
- The authors indicated that this approach was "...an economical and simple assay to follow and characterize membrane receptor dynamics..." and that the use of epifluorescence microscopy (EM) was possible. However, in their descriptions, the authors used confocal microscopy and some related techniques such as image stacking (not really accessible in EM). The authors should describe the use of EM in this approach.

Working in Z-stack and quantifying fluorescence in each slice, it is important to have a good 3D sectioning. We achieved it using structured illumination technology which is less expensive and time-consuming than a confocal laser microscopy. Alternatively a deconvolution algorithm (plugins for Fiji or DeconvolutionLab and DeconvolutionLab2 (EPFL) (https://bigwww.epfl.ch/deconvolution/) can be used to analyze fluorescent stack image series . See lines 196-202 and Table 1

- The number of cells analyzed was not reported. The number of experiments was also not reported. These items should be reported.

We apologize for forgetting to include the number of analyzed cells in each experiment and the number of experiments carried out. For each experiment, repeated three times, 20 cells were analyzed at each time point. We added this information in the Expected Results section (see linse 161-162).

- How was the percentage of positive areas determined? Image analysis? Software used? procedure?

All the information regarding image quantification is reported in Supporting information: Step 5, Image quantification and data analysis (day 5). Briefly, for each experiment images were acquired with the same

UNIVERSITÀ DEGLI STUDI DI BRESCIA
DIPARTIMENTO DI MEDICINA MOLECOLARE E TRASLAZIONALE
SEZIONE DI BIOTECNOLOGIE
Prof.ssa Stefania Mitola
Tel. 030 3717312 - E-Mail: stefania.mitola@unibs.it

exposure time. Then, Z-stacks were analyzed with Fiji software. After defining a threshold, we drew a ROI for each cell in which we quantified the number of fluorescence-positive pixels in every slice of the Z-stack. Finally, we calculated the percentage of the receptor present in every slice to visualize its distribution.

- The authors do not discuss other methods to study receptor dynamics. This should be discussed. The advantages and limitations of this new approach should also be discussed.

We thank both Reviewers for the suggestion of deepening advantages and limitations of the methods currently available to study receptor dynamics on cell membranes. To address it, we decided to include a Table in Discussion section (Table 1, line 222) describing positive and negative aspects of the proposed protocol compared with the other two main methodologies: FRAP and SPT.

- The authors cited reference 17 for analysis of VEGFR and other receptor expression, but this reference did not use the same method. This should be discussed.

*In the manuscript we refer to the papers by Damioli et al. (*doi: 10.1038/s41598-017-16786-4.*) and Ravelli et al. (*doi: 10.1161/ATVBAHA.115.306230*I) in which this protocol was used. In those publications, however, we focused our attention only on the quantification of receptors (VEGFR2 and Beta3 integrin) recruited in close contact with the immobilized factors. Here we analyzed the distribution, in time, of the entire pool of receptor.*

- The authors do not indicate whether the internalization of the receptors could be detected with this method ? or not.

We added a comment on the possibility of detection receptor internalization in lines 218-222.

Reviewer #2:
The authors provide an alternative cost effective method for visualizing receptor dynamics in cell membranes. The protocol is well-described, providing clear step-by-step instructions for conducting the experiments. There are few suggestions that would help to improve the manuscript:

1. The introduction needs to be more general rather than focusing on one type of membrane receptors.

We introduced the different classes of membrane receptors in the introduction section (see lines 32-36) , Then we focused only on one pass transmembrane receptors. Although we have no reason to think that the described protocol cannot be used to follow all kinds of receptors, we have alway applied it to analyze the dynamics of single-pass receptors (e.i tyrosine kinase and integrins).

2. Figure 1: Experimental layout - the steps shall be described in details with graphical/pictorial representation of steps making it easier for the readers to follow.

Following the reviewer's suggestion we included in the revised version (Fig 2) a cartoon which recapitulates the steps from the coverslip flipping to image quantification. Fig 2: Cartoon of steps 3-5 of experimental flow chart (see lines 89, 147 and Fig2).

3. Authors should incorporate the practical problems faced and troubleshooting they performed.

UNIVERSITÀ DEGLI STUDI DI BRESCIA
DIPARTIMENTO DI MEDICINA MOLECOLARE E TRASLAZIONALE
SEZIONE DI BIOTECNOLOGIE
Prof.ssa Stefania Mitola
Tel. 030 3717312 - E-Mail: stefania.mitola@unibs.it

The practical problem and the critical steps were included into *DOI: dx.doi.org/10.17504/protocols.io.rm7vzj8z8lx1/v1 (Private link for reviewers: https://www.protocols.io/private/46322724FDB111EEB7ED0A58A9FEAC02 to be removed before publication.)*

4. Lastly, it would be good to add a table showing the limitations and advantages of the existing techniques including the one described by the authors.

We included limitations and advantages in Table 1 (Advantages and disadvantages of microscopy techniques for the study of molecule relocation) and described in the Discussion section of the revised version. See Table 1, line 222 .

Sincerely

Prof.ssa Stefania Mitola

Viale Europa 11
25123 Brescia
BS (Italy)

Tel + 39 030 3717.1
dmmt@cert.unibs.it
www.unibs.it

Servizi Amministrativi
Tel +39 030 3717401-405
segreteria.dmmt@unibs.it
